# Investigating the effectiveness of the *Trigonella foenum-graecum* L. (fenugreek) seeds in mild asthma: a randomized controlled trial

**DOI:** 10.1186/s13223-018-0238-9

**Published:** 2018-05-02

**Authors:** Majid Emtiazy, Laleh Oveidzadeh, Minoo Habibi, Leila Molaeipour, Daryush Talei, Zahra jafari, Mahmoud Parvin, Mohammad Kamalinejad

**Affiliations:** 1 Department of Persian Medicine, The School of Persian Medicine, Shahid Sadoughi University of Medical Sciences, Ardakan, Yazd Iran; 2 Internal Medicine, Pulmonary Ward, Labbafi Nezhad Hospital, Shahid Beheshti University of Medical Sciences, Tehran, Iran; 3 Department of Epidemiology, Pasteur Institute of Iran, Tehran, Iran; 4 Medicinal Plants Research Center, Shahed University, Tehran, Iran; 5 Pathology Department, Labbafinejad Hospital, Shahid Beheshti University of Medical Sciences, Tehran, Iran; 6 Department of Pharmaceutics, Faculty of Pharmacy, Shahid Beheshti University of Medical Sciences, Tehran, Iran

## Abstract

**Background:**

Asthma is one of the important chronic diseases. The asthma prevalence is increasing in last decades. Despite the presence of good controller drugs like corticosteroids, about 60% of asthmatic patients use alternative medicine. This study was done to determine the effectiveness of *Tregonella foenum graceum* (fenugreek) seeds in mild asthma.

**Methods:**

It is a double blind trial with placebo effect. One of the ancient prescriptions from Persian Medicine was selected. The participants were divided to three groups randomly. On group received fenugreek syrup one received honey syrup and the third received placebo. Duration of treatment was 4 weeks. Quality of life, Lung function tests and IL-4 levels were evaluated before and after treatment.

**Results:**

From 90 participants to study 79 completed the process. After study there was significant increase in quality of the life and lung function tests and IL-4 levels in fenugreek and honey groups.

**Conclusion:**

FEV1 level was improved more than 10% in fenugreek group. Treatment was well tolerated. No serious side effects were reported during the study. The aqueous extract of fenugreek seeds appears to be effective and safe in treatment of mild asthma.

*Trial registration* The study was recorded with the Iranian Registry of Clinical trials [http://www.irci.ir], registration code: IRCT2016011325991N1

**Electronic supplementary material:**

The online version of this article (10.1186/s13223-018-0238-9) contains supplementary material, which is available to authorized users.

## Background

Asthma is characterized by variable degrees of airway obstruction. This obstruction is usually reversible and leads to symptoms of dyspnea and wheezing. The increasing prevalence of asthma has led to high costs and burden of disease for the general population and government. More than 300 million people worldwide have asthma [1, 2]. Asthma is affecting 18.9 million adults only in USA [3]. The reported prevalence in Iran varies from 5 to 15% [4].

Widespread uses of inhaled corticosteroids have improved lung function tests but concerns about their side effects are increasing [5], so further researches is needed. There is also an increasing and significant interest in using herbal medicine. In one report, about 60% of asthmatic patients used alternative medicine [6].

Traditional Persian Medicine (TPM) one of an ancient traditional medicines, recommended prescriptions that are still use in Iran and nearby countries. One of these prescripts is based on *Trigonella foenum*-*graecum* for asthma treatment, with common name *fenugreek.* It is an annual plant, 10–50 cm high. Its medicinal parts are ripe, dried seeds. The main growth regions are in Southern France, Turkey, Northern Africa, India, and China [7]. According to Flora Iranica, more than 32 species of this plant have been found in central regions of Iran [8].

*Trigonella foenum*-*graecum*, [HULBAH in TPM], is used as an appetite stimulant, lung tonic, and chest wall analgesic, also enhances breathing and lung secretion, clears the voice, and induces menstruation [9–11]. In recent studies, *T. foenum*-*graecum* seeds have been used for their antidiabetic and cholesterol-lowering effects [12–16]. They also have anti-inflammatory and antioxidant effects [17–19]. The most significant antioxidant activity in this remedy comprises polyphenols [20]. In animal studies, *T. foenum*-*graecum* extract reduced edema in a dose-dependent manner [21]. The seeds also consist of 50%, fiber which is mucilaginous [22]. Some of the active components in *T. foenum*-*graecum* are alkaloids (0.36%), saponins, steroidal sapinogens (0.1 to 2.2%), flavonoids, unsaturated fatty acids (6–10%), fiber, and amino acids. Other components include coumarin, lipids, vitamins, minerals, mucilage (28%), and proteins (22–25%).

Despite extensive use of *T. foenum*-*graecum* in TPM, there has been no placebo-controlled trial for its effect on asthma treatment. Therefore, this study investigated the efficacy of the seed extract of *T. foenum*-*graecum (Fenugreek)* in adults with mild asthma.

## Methods

### Participants

This study is a preliminary investigation of effectiveness and safety of *T. foenum*-*graecum* in mild asthma patients. The study was conducted in the respiratory polyclinic of Shahid Labaffi Nezhad Hospital in Tehran, Iran between May 2016 and March 2017.

### Inclusion criteria

Patients aged 20–70 with mild asthma diagnosed by a physician according to Global Initiative for Asthma guidelines [5], and who signed a consent form were included in the study. Use of any complementary or alternative medicine during the study was prohibited.

### Exclusion criteria

Patients who used monoamine oxidase inhibitors were excluded from the study. Those with any allergic reaction to fenugreek, peanuts, or soybeans were also excluded. Additional exclusion criteria were any severe disease, seasonal allergy, pregnancy and breast feeding, hypothyroidism, use of anticoagulant drugs, diabetes, and addiction to opiates. Those with a history of any side effects to medicine used in the study were also excluded. Each participant was permitted to discontinue the study for any reason at any time.

### Ethical considerations

The study design was in compliance with the guidelines of the Declaration of Helsinki. The study was approved by the ethics committee of Yazd Shahid Sadoughi University of Medical Sciences with registration number: IR.SSU.REC.1396.53.

The study was recorded with the Iranian Registry of Clinical trials[http://www.irci.ir], registration code: IRCT2016011325991N1.

We obtained informed consent from all participants.

### Study design

This study was a placebo-controlled double blind clinical trial. The study began in the respiratory polyclinic of Shahid Labaffi Nezhad Hospital. It had three parallel groups. One group received fenugreek seed syrup, the second received honey syrup, and the third received placebo syrup. All patients received their medication (B_2_ agonists) according to the definition of mild asthma [5].

The intervention used in this study was an aqueous extract of *T. foenum*-*graecum* (fenugreek; shanbalileh or hulbah) seeds based on TPM formula. Preparation of syrups was done in the herbal medicine laboratory of the Shahid Beheshti School of Pharmacy, Tehran, Iran. The extract was produced from whole dry fenugreek seed purchased from a local herbal medicine market. The herbs were approved by the herbarium of the Shahid Beheshti School of Pharmacy (No. 8065). One liter of boiled (100 °C) water was added to 100 g of whole dry seeds and kept in a closed container in the laboratory for 4 h. The extract was filtered and concentrated using the Ben Murray method. Honey solution 50% was added to the extract to obtain 50 g of extract in 100 ml of syrup. Honey was prepared from *Rayehe Khansar* Company (Reg. No: 143442 Natural Honey Product of Iran). Honey syrup also contained a 50% solution. Placebo was prepared using a pharmacopoeia, with simple syrup based on a 50% sugar solution; approved color additives were used for the same appearance as the *T. foenum*-*graecum* syrup. We used the SPIRIT 2013 check list to design of this study (Additional file 1)

### Intervention

Patients were alternately and equally allocated to three groups: fenugreek group, honey, and placebo group. Subjects consumed 10 ml of the syrup twice daily for 4 weeks. This duration was used regarding to previous herbal investigations in asthma treatment [23]. The herb dosage was determined by referring to the PDR of Herbal Medicine [7]. No nutrition advice or restrictions were suggested.

### Measurements

Two questionnaires were used in this study. The St. George’s Respiratory Questionnaire evaluates health impairment in asthma patients, based on three items: an activity score that measures impairment of daily physical activity, an impact score that covers a wide range of disturbances of psychosocial activity, and symptom recall for a preceding period ranging from 1 month to 1 year; the total score shows overall health status in asthmatic patients. The score is expressed as a percentage: 100% indicates the worst possible health status and 0% indicates the best possible health status [24]. We used the Persian validated version of this questionnaire [25]. The second questionnaire was the asthma control test (ACT), with scores ranging from 5 to 25. Higher scores indicate better disease status. Scores of 20–25 indicate well-controlled asthma, 16–20 indicates fair control, and 5–15 indicates poor control. The ACT includes four symptom questions plus a patient self-assessed level of control. The minimum clinically important difference is three points [26]. We used the Persian validated version of ACT [27].

Blood samples were taken to determine any organ abnormalities caused by our therapy. To assess lung function, the forced expiratory volume in 1 s (FEV1), peak expiratory flow (PEF), and maximal expiratory flow rate 25–75% (MEF_25–75_) were measured using a respiratory laboratory system (nSpire Health GmbH; Oberthulba/Germany) before and after the study.

Serum interleukin-4 (IL-4) (as a Th2 profile) levels were determined using an enzyme-linked immunosorbent assay (IL-4 kit; IBL international GMBH, Hamburg, Germany).

Participants included asthma patients that their disease were diagnosed in the prior year. After screening for eligibility, consent forms and the two questionnaires were completed. Lung function tests and venous blood samples were obtained for all participants before and after treatment. Participants were randomized when visiting the respiratory clinic and were allocated to the control arm or to the intervention arm by a researcher who did not know the content of the syrup bottles. The syrups were packed and alphabetically labeled in the same opaque and sealed bottles. This allocation was performed by an independent investigator at Shahid Beheshti Pharmacy University. Participants were followed for 4 weeks and then scheduled an appointment to visit the specialist and complete the questionnaires, lung function test, and blood tests. Patient compliance was checked every week by telephone. A telephone number was provided for any further questions. Subjects could contact the number at any time.

### Sample size

**Fig. 1:**
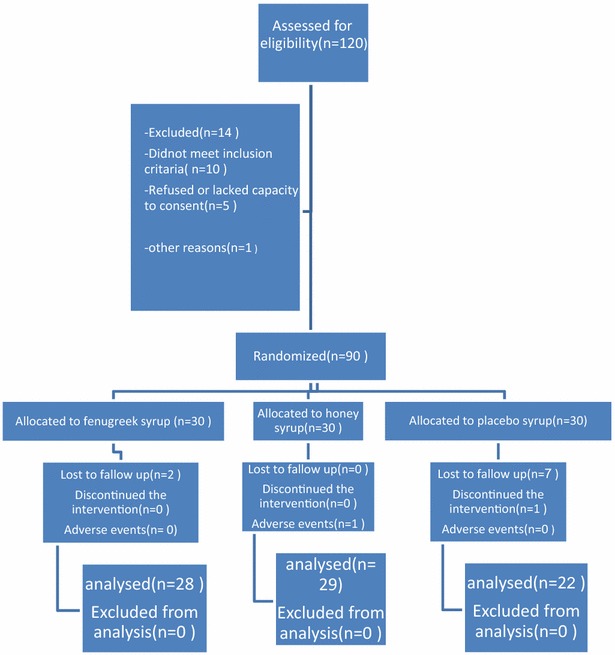
The consort flow chart

Total 120 patients with mild asthma were randomly assigned, from this 90 were in compliance with eligibility criteria. They were allocated to three groups. One group received fenugreek syrup, one received honey, and one received placebo. The consort flow diagram of the study is shown in Fig. 1. We collected 20 subjects from each group by random selection to measure IL-4 in their blood samples before and after treatment. We considered the type 1 error rate and study power to be 5 and 80% to detect a statistically significant difference in three groups.

### Statistical analysis

The data for participant age, gender, weight, and height were described as mean ± standard deviation (SD). Comparisons between three groups were performed with the independent sample t test and Chi square (or Fisher’s exact) tests. Data were analyzed using SPSS version 21 (SPSS Inc., Chicago IL, USA). P values ≤ 0.05 were considered statistically significant. Adverse events during therapy were recorded for reporting at the end of the study.

## Results

### Patient characteristics

At the end of study 79 (49 women and 30 men) completed the study. There were 22 in the placebo group, 28 in the fenugreek group, and 29 in the honey group that completed the study. At the start of the study, there were no significant differences between groups with regard to gender, FEV1, PEF, MEF_25–75_, and the two questionnaire scores (Table 1).

**Table 1:** Basic demographic characteristics of participants included in the trial

Parameters	Fenugreek	Honey	Placebo	P value
Sex (female/male)	16	22	11	0.57
	12	7	11	
Age	41.1 ± 13.6	45 ± 12.9	41.6 ± 12.5	0.38
St. George	53.22 ± 2.84	52.75 ± 1.95	46.65 ± 3.73	0.11
FEV1%	90	80	93	> 0.05
ACT	16.63 ± 1.92	16.45 ± 3.98	16.27 ± 2.53	> 0.05

After matching the effect of possible confounding variables (age) the total score on the ACT in the three groups indicated fair control. Improvement in asthma symptom control (ACT questionnaire) in the fenugreek and honey groups was significant by the end of the study (16.63–20; P < 0.01 in the fenugreek group, and 16.45–20.36; P < 0.01 in the honey group). The placebo group showed no significant difference (16.27–17).

The St. George Questionnaire scores showed in Table 2. Regarding to results we used the Bonferroni test to compare differences. There was a significant higher mean difference in total St. George score in the fenugreek group in comparison with the placebo group (P < 0.001) and between the honey and placebo groups (P < 0.001); there was also a significant difference between the honey and fenugreek groups (P = 0.002).

**Table 2:** The St. George’s result of participants before and after treatment

Parameters	Before study ± SD	After study ± SD	P value
Activity	Fenugreek: 47.9 ± 3.68 Honey: 56.41 ± 2.4 Placebo: 47.76 ± 4.73	Fenugreek: 36.51 ± 2.37 Honey: 47 ± 2.67 Placebo: 51.19 ± 4.65	< 0.01
Impact	Fenugreek: 50.73 ± 2.74 Honey: 45.74 ± 2.16 Placebo: 41.28 ± 4.58	Fenugreek: 23.65 ± 1.41 Honey: 27.75 ± 1.63 Placebo: 36.65 ± 3.08	< 0.01
Symptom	Fenugreek: 62.89 ± 4.59 Honey: 59.63 ± 2.49 Placebo: 53.38 ± 2.61	Fenugreek: 41.1 ± 2.44 Honey: 43.93 ± 2.23 Placebo: 48 ± 3.61	< 0.01
Total score	Fenugreek: 53.22 ± 2.84 Honey: 52.75 ± 1.95 Placebo: 46.65 ± 3.73	Fenugreek: 30.27 ± 1.6 Honey: 36.24 ± 1.8 Placebo: 42.91 ± 3.57	< 0.01

Spirometry parameters of participants before and after treatment showed in Table 3.

**Table 3:** The Spirometry’s result of participants before and after treatment

Spirometry parameters	Before treatment ± mean SD	After treatment ± mean SD	P value
FEV1%	Fenugreek: 90 ± 10.99 Honey: 80 ± 23.11 Placebo: 93 ± 12.32	Fenugreek: 100 ± 12.45 Honey: 87 ± 19. 10 Placebo: 94 ± 10.14	0.02
FEV1/FVC	Fenugreek: 0.82 ± 3.54 Honey: 0.81 ± 7.05 Placebo: 0.86 ± 2.34	Fenugreek: 0.89 ± 11.66 Honey: 0.85 ± 10.60 Placebo: 0.85 ± 6.73	0.06
PEF	Fenugreek: 67 ± 5.75 Honey: 61 ± 18.29 Placebo: 73 ± 10.27	Fenugreek: 80 ± 12.70 Honey: 72 ± 17.33 Placebo: 74 ± 11.68	0.03
MEF_25–75_	Fenugreek: 87 ± 18.94 Honey: 72 ± 24.15 Placebo: 91 ± 18.13	Fenugreek: 101 ± 14.10 Honey: 84 ± 23.11 Placebo: 91 ± 19.81	< 0.01

Regarding to results FEV1%, The forced expiratory volume/forced vital capacity (FEV/FVC) ratio, PEF and MEF_25–75_ values showed significant improvement at the end of the study in the fenugreek group and honey group but not in the placebo group (see Table 3).

**Fig. 2:**
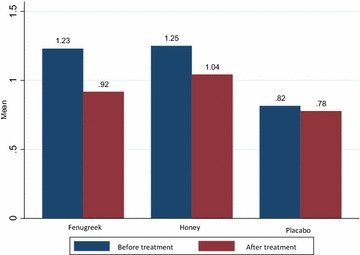
Mean IL-4 (pg/ml) of groups before and after treatment

Reduction in IL-4 level was observed in the fenugreek (P < 0.001) and honey groups (P < 0.001), but no significant reduction was observed in the placebo group (Fig. 2). To compare the differences between the groups we used the Bonferroni test. According to the test there were significant differences between the mean levels of IL-4 in the fenugreek group in comparison with the honey (P = 0.002) and placebo (P < 0.001) groups. Significant differences were also observed between the honey and placebo groups (P < 0.001).

### Safety of treatment

Participants tolerated the selected treatment. Neither group needed emergency care. One patient in the honey group left the study because of diarrhea. No abnormal findings were observed in para clinic data. Two women in the fenugreek group used more pads during menstruation but no serious adverse effects were observed in any group.

## Discussion

This study is a preliminary investigation of the efficacy and safety of *T. foenum*-*graecum* (fenugreek) seed extract as add-on treatment in mild asthma. The fenugreek syrup was prepared in honey solution as TPM formula. The aqueous extract of fenugreek seeds improved the lung function tests and Quality of Life in patients with mild asthma significantly in compare with honey and placebo syrup. The use of fenugreek seed extract led to a 10% increase in FEV1 and FEV1/FVC levels, in addition to a significant decrease in serum cytokine IL-4 levels.

Based on few side effects, the study findings suggest that the aqueous extract of fenugreek seeds can be used in mild asthma treatment. To the best of our knowledge, this is the first study to compare fenugreek efficacy with placebo.

*Trigonella foenum*-*graecum* was selected among TPM prescriptions [9–11]. There are many different formulas in ancient medical textbooks for asthma treatment. We selected a simple combination to observe its effects or side effects more closely during the study.

According to TPM, fenugreek is a lung tonic, and can facilitate lung secretions [9–11]. In recent researches, fenugreek is recognized for its antidiabetic [12–16], anti-inflammatory, and antioxidant effects [17–19].

Although there is no direct study about fenugreek and asthma treatment, some of its components have been shown to have special efficacy in treatment of asthma. Among the important components are flavonoid; a group of low-molecular-weight polyphenols, which are effective in asthma treatment to some extent. In various studies, fenugreek was found to have antioxidant effects due to the ability of flavonoids to inhibit lipid peroxidation and protect the airways against oxidative stress [28, 29]. Flavonoids also inhibit the activation of basophils and mast cells [17].

Along with the antioxidant effect of fenugreek, quercetin is a flavonoid that can inhibit Charcot–Leyden crystals and eosinophil cationic proteins, which are involved in asthma pathogenesis [30, 31]. It also has ability to reduce the damage caused by oxidative agents [32, 33].

Flavonoids can also inhibit some steps in angiogenesis, such as cell migration and microcapillary tubule formation [34, 35]. As shown in previous studies; vascular changes have an important role in asthma pathogenesis [36].

The anti-inflammatory effects of fenugreek seeds have also been demonstrated [37].

Another possible mechanism of action of fenugreek is due to the mucilage component of seeds (28%), which may facilitate lung secretions and improve asthmatic cough [9–11].

Regarding to importance of the relationship between cytokine imbalance (Th1/Th2) in both atopy and asthma expression we selected IL4 as a Th2 profile [38]. In this study IL4 levels significantly decreased after the treatment process in both fenugreek and honey group, of course the effectiveness of fenugreek was more than honey. IL4 divided from T lymphocytes and mast cells could lead to IgE synthesis and airway inflammation [39, 40]. Many studies determined the effect of IL4, IL5 and IL13 in asthma pathogenesis there for making balance between TH1 and TH2 cytokines might be helpful in asthma management [41].

Fenugreek has a wide dose range. In one human study, 25 g of fenugreek seed powder daily was well tolerated and without serious side effects [40]. Acute toxicity values(LD50) documented for fenugreek are 5 g/kg (in rats, oral) and 2 g/kg (in rabbit, dermal) from alcoholic seed extract [42]. The dosage selected in this study was based on PDR for herbal medicine [7].

Despite the effectiveness of fenugreek seeds demonstrated in this study, specific mechanisms for its efficacy remain unknown, and further studies with a larger sample size are needed.

The next important issue in this study was the role of honey. As the results showed, honey syrup could improve the Quality of Life for asthmatic patients, in addition to improving lung function tests and reducing the level of IL-4. Although the effect of honey syrup was less than that of fenugreek syrup in all items, the combination of honey and fenugreek appears to have increased the efficacy of formula.

Many TPM prescriptions use honey as a preservative, but honey also has many benefits in lung diseases [9–11]. Recent studies showed the antioxidant effects of honey [43, 44].

However, honey contains flavonoids and can inhibit the growth of certain microbes because of the concentration of sugar and low pH [45, 46]. Research has also shown the anti-inflammatory effects of natural honey [47].

In conclusion the aqueous extract of *T. foenum graceum* (Fenugreek) in honey based syrup showed acceptable effect in our preliminary study as an add-on treatment in mild asthma.

Due to good results and well tolerance of the fenugreek syrup, this remedy could be suggested as an adjuvant therapy in mild asthma treatment.

## Study limitation

Despite the demonstration of effectiveness of fenugreek seeds in mild asthma treatment, this study also had some limitations. An important limitation was the small sample size. A short duration of follow-up is another limitation; longer studies could reveal more side effects or even more efficacy of fenugreek in asthma treatment. Another limitation was the need to prepare syrups with the same color, taste, and smell; however, the pharmaceutical worker did his best to achieve similarity.

Because of the wide use of corticosteroids in asthma treatment, we recommend further studies to compare the effectiveness of fenugreek syrup with corticosteroid drugs.

## Additional file

**Additional file 1.** **SPIRIT 2013** CHECKLIST is recommended to address items in study protocol and related documents.
